# A novel validated assay to support the discovery of new anti-malarial gametocytocidal agents

**DOI:** 10.1186/s12936-016-1429-9

**Published:** 2016-07-22

**Authors:** Noemí Bahamontes-Rosa, María G. Gomez-Lorenzo, Joël Lelièvre, Ane Rodriguez Alejandre, María Jesus Almela, Sonia Lozano, Esperanza Herreros, Francisco-Javier Gamo

**Affiliations:** Tres Cantos Medicines Development Campus, Diseases of the Developing World, GlaxoSmithKline, Tres Cantos, 28760 Madrid, Spain

**Keywords:** *Plasmodium falciparum*, Real time PCR, qPCR, Gene expression, mRNA, Gametocyte, Transmission

## Abstract

**Background:**

Drugs that kill or inhibit *Plasmodium* gametocytes in the human host could potentially synergize the impact of other chemotherapeutic interventions by blocking transmission. To develop such agents, reliable methods are needed to study the in vitro activity of compounds against gametocytes. This study describes a novel assay for characterizing the activity of anti-malarial drugs against the later stages of *Plasmodium falciparum* gametocyte development using real-time PCR (qPCR).

**Methods:**

Genes previously reported to be transcribed at the different sexual stages of the gametocytogenesis were selected for study and their mRNA expression was measured in a gametocytogenesis course by qPCR. Genes mainly expressed in the later stages of gametocyte development were used as a surrogate measurement of drug activity. To distinguish between cidal and static drug effects, two different experiments were performed in parallel, one with constant drug pressure throughout the experiment (144 h), and another in which the gametocyte cultures were exposed to the compound for only 48 h.

**Results:**

Four *P.falciparum* genes coding for proteins Pf77, ROM3, Pfs25, and Pfg377 with transcription specific for late-stage gametocyte development were identified. The in vitro anti-malarial activity of compounds against such gametocytes was assessed by measuring mRNA levels of these genes using qPCR. The assay was validated against standard anti-malarial drugs (epoxomicin, dihydroartemisinin, chloroquine, thiostrepton, and methylene blue) and compounds from the GSK compound library with known anti-gametocyte activity.

**Conclusions:**

This study describes a novel assay for characterizing the activity of anti-malarial drugs against the later stages of *P. falciparum* gametocyte development using qPCR in genetically unmodified parasites. The method described is a reliable and user-friendly technique with a medium throughput that could be easily implemented in any laboratory.

**Electronic supplementary material:**

The online version of this article (doi:10.1186/s12936-016-1429-9) contains supplementary material, which is available to authorized users.

## Background

Malaria remains one of the most widespread infectious diseases and a major global health problem. In 2015, there were an estimated 214 million malaria cases, with 438,000 deaths [1]. Malaria is caused by protozoan parasites of the genus *Plasmodium*. Parasite asexual stages cause the clinical symptoms of malaria, and the sexual stages (gametocytes) allow transmission of the parasite from human to mosquito.

Transmission blocking is a key strategy highlighted in the Malaria Eradication Research Agenda (malERA) [2]. In the absence of an effective transmission-blocking vaccine, chemotherapy remains a cornerstone of current interventions [3]. Although many of the classical anti-malarial drugs are active against early gametocyte stages, mature gametocytes are unresponsive to these drugs. Currently, only primaquine, an 8-aminoquinoline, is approved for clinical use as a transmission-blocking agent. However, primaquine has toxicity issues, causing haemolysis in glucose-6-phosphate dehydrogenase (G6PD)-deficient individuals [4, 5].

Future anti-malarial treatments should ideally target both the rapidly replicating asexual stages and the less metabolically active, non-replicating, mature gametocytes. Recently, several in vitro assays have been developed that allow the investigation of drug activity during gametocytogenesis and against sexual forms [6–12]. However, there remains a need to confirm the cidal effect of new compounds on mature gametocytes prior to validating their transmission-blocking potential ex vivo using the standard membrane-feeding assay (SMFA).

Detecting and quantifying specific mRNA levels directly reflects gene expression and is, therefore, a hallmark of viable cells. A real-time PCR (qPCR) assay was previously reported, focused on asexual stages, that allows classification of compound anti-malarial activity as ‘cidal’ or ‘static’, using mRNA expression levels as a surrogate of parasite viability [13]. In the asexual stage assay, a drug was considered ‘static’ if the active growth of parasites was arrested in its presence, but resumed once drug was removed from the medium. A drug was considered ‘cidal’ if parasite growth was not resumed following the removal of drug pressure, evidenced by a significant reduction in mRNA expression levels (more than 100 times *vs* untreated controls). In the context of gametocytes, cidal compounds are those that produce irreversible changes in the parasite that are sustained once the compound is removed; ‘static’ compounds are those that exert a deleterious effect only when the compound is present. However, because of the low metabolism of gametocytes, mRNA levels will not recover.

The objective of this study was to further develop the above assay to allow quantitative analysis of the levels of late-stage gametocyte-specific mRNAs. The robust identification of genes specific expressed during the later stages of gametocyte development, different from those detected in asexual forms and young gametocytes, was therefore critical to support the use of qPCR to distinguish molecules active against late-stage gametocytes*. Plasmodium falciparum* gametocytogenesis lasts about 8–12 days, and has been traditionally divided into five distinct morphological stages corresponding to different phases of maturation [14, 15]. Microarray experiments have demonstrated fluctuating mRNA levels for many genes through the parasite lifecycle, discerning the expression of several genes as stage specific [16–19], with some confirmed by qPCR [20–22].

In this study, a snapshot of the expression profile of the 12 best-described candidate genes for identifying mature gametocytes was generated. Having identified genes expressed specifically during the later stages of gametocyte development, the methodology already established to classify compounds with cidal activity for asexual stages could be extended to identify those with gametocytocidal activity. This assay was validated using commercially available anti-malarial drugs plus compounds with proven transmission-blocking activity selected from the corporate GSK compound collection [23].

## Methods

### Chemicals

All commercially available and GSK compounds previously characterized as anti-malarial agents were dissolved in H_2_O or dimethyl sulfoxide (DMSO, SIGMA, Steinheim, Germany) at variable stock concentrations. All stock solutions were kept at −20 °C.

### Parasite culture

*Plasmodium falciparum* chloroquine-sensitive strain 3D7A was obtained from the Malaria Research and Reference Reagent Resource Center (MR4) and was maintained in continuous culture at 4 % haematocrit using fresh AB+ erythrocyte concentrates provided by the Spanish Red Cross blood bank of Madrid, Spain. Gametocyte production and maturation followed the protocol published by Lelievre et al. [24]. Two independent gametocytogenesis courses were performed, maintaining cultures for 15 and 30 days. Both parasitaemia and gametocyte maturation were monitored by thin blood smears stained with 10 % Giemsa (Merck) in phosphate buffer pH 7.2. During the time course, 200 µl of total parasite culture (12 % haematocrit) was harvested daily, pelleted down and then stored at −80 °C for subsequent qPCR analysis. Sampling was performed in duplicate.

As an alternative to the standard procedure described [24], gametocytes were cultured for 20 days adding 50 ng/ml bistratene A to the cultures twice a day from day 4 to remove the asexual stages earlier. One millilitre of culture at 12 % haematocrit was pelleted on days 1, 4, 7, 15, and 20 and then stored at −80 °C for analysis.

### Cidal vs static activity assay

**Fig. 1:**
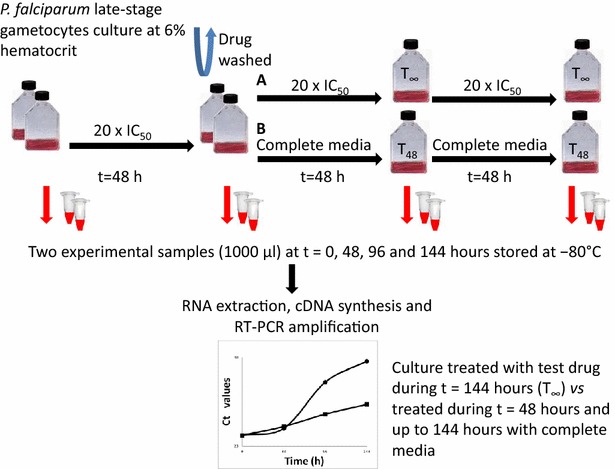
Scheme showing the cidal *vs* static in vitro activity assay against stage V *Plasmodium falciparum* gametocytes. Gametocytes were incubated for 48 h with 20 × IC_50_. After drug was washed out, the parasites were cultured for 96 h with complete culture medium with drug (*A*) or without drug (*B*) to evaluate the reversibility of compound effects. Two samples at t = 0, 48, 96, and 144 h were taken for RNA extraction, cDNA synthesis and qPCR amplification. Comparison of mRNA levels of treated *P. falciparum* cultures upon removal of the drug was used to assess the cidal *vs* static activity

The in vitro cidal or static anti-malarial activity of a compound against the later stages of gametocyte development at day 15 was assessed by measuring mRNA levels in *P.falciparum* over 144 h following drug treatment (Fig. 1). Drug activity assays were performed once in two independent cultures in T-75 flasks (Corning, NY, USA). Late-stage gametocytes were incubated with drug and 6 % haematocrit over 48 h. Compounds were used at a concentration of a minimum of 20-fold their IC_50_ against mature gametocytes (20 × IC_50_), with the exceptions of methylene blue, tested at ten times its IC_50_, epoxomicin, tested at two times its IC_50_, and chloroquine, tested at 5 µM corresponding to 125 times the asexual IC_50_; concentrations are specified in Table 1. IC_50_ values were obtained from Lelievre et al. [24]. To remove drug from the medium, parasite cultures were harvested and washed two times with 25 ml of RPMI. After drug removal, the parasites were cultured for a further 96 h with complete culture medium either without or with drug, at the same concentration as the initial incubation, to evaluate the reversibility of the compound effect. Medium was changed daily and two samples of 1000 µl of culture at 0, 48, 96, and 144 h were taken, centrifuged and immediately stored at −80 °C for further analysis. An untreated culture was used as a positive control.

**Table 1:** Characteristics of the anti-malarial compounds tested in the assay

Compound^e^	Structure	P.f. gametocytes IC_50_ (µM)	TOX50 (µM)^b^	Assay concentration (µM)^c^
Chloroquine^d^	–	23.47	51.84	5.00
Methylene blue	–	0.49	6.52	5.00
Dihydroartemisinin	–	3.56	>50	71.20
Epoxomicin	–	0.0005	0.003	0.001
Thiostrepton	–	0.90	–	18.00
TCMDC-134278		>5	19.95	NT
TCMDC-136869		1.66	12.59	NT
TCMDC-125520		0.0098	12.80	0.20
TCMDC-123475		0.0098	>25	0.20
TCMDC-125133		0.54	>50	10.76
TCMDC-125769		0.04	>25	0.86
TCMDC-125114		0.13	>50	2.60

^a^IC_50_ values measured in the laboratory for 3D7A *P. falciparum* mature gametocytes with the ATP bioluminescence assay [24]

^b^Cytotoxicity data using the short-term resazurin-based reductase assay [24]

^c^Working concentrations were 20 × IC_50_ if that was below the TOX_50_

^d^Working concentration of chloroquine was 5 µM corresponding to ×125 the asexual IC_50_

^e^GSK compounds were published in Ref. [23]

### RNA extraction and cDNA reverse transcription

For RNA extraction, RNeasy Plus mini kit (Qiagen, Hilden, Germany) was used according to the standard protocol supplied by the manufacturer. cDNA synthesis and SYBR green qPCR were performed in duplicate following the protocol already published by Bahamontes-Rosa et al. [13]. Melting curve analysis was always performed at the end of each assay as a specificity control.

### Primer design

All genes included in the study encode proteins described a priori as specific for gametocyte stages. mRNA gene sequences were retrieved from PlasmoDB database and used as templates to design qPCR primers using the Primer Express Software v.1.5 (Applied Biosystems) (Table 2).

**Table 2:** Differentially expressed genes in the parasite life cycle, and primers for qPCR

Gene ID^a^	Description	Primers^b^
Housekeeping gene		
18S rRNA	18S ribosomal RNA (PF3D7_0112300)	AATAACAATGCAAGGCCAATTT CTGCAACAATTTTAATATACGC
Young gametocytes		
PFD0310w	Sexual stage-specific protein precursor (Pfs16)	AGTTCTTCAGGTGCCTCTCTTCA AGCTAGCTGAGTTTCTAAAGGCA
PF14_0748	Exported protein (PHISTa), unknown function (Pfg14-748)	CTTATGTGCTGAATTTTGTGTTATGGT TGTAAACTCTGATTTGGCCACACT
PF13_0011	Gamete antigen 27/25 (Pf 27\25)	CATGAAACACATGCCCCTCTCT GCTACAGGCATGAAACTCAATATCC
PF14_0744	Exported protein, unknown function (Pfg14-744)	TTATGTATAATGGCTCTGTTGACGG GGCTTCTCGACTTCCTCGAA
PFL0795c	Male development gene 1 (Pfmdv-1/peg3)	TTTGAGACATTTGAACAAGCTTTACA GCATTTCCGGATTTGTTATTTTC
PF10_0164	Early transcribed membrane protein 10.3 (ETRAMP10.3)	TGCTGCTGTTGCTTTGGCTA CCTCAGAGTCGGATCCATCATT
Mature gametocytes		
PFF1035w	(Pf77)	GGAAGACAAAAAACACTGCACATTA TTCAAACCATCGTCCTCTTTTTC
PFL2405c	Female specific gametocyte-specific (Pfg377)	TGTTCTTTTTCATATCGTCTATCTTCCT TTGCTTTCCTTAAGATGTTTAATGATG
PF10_0303	25 kDa ookinete surface antigen precursor (Pfs25)	GACTGTAAATAAACCATGTGGAGA CATTTACCGTTACCACAAGTTA
PFL2510w	Chitinase (pfcht1)	TCGAGCACGACCAGGTGAA CCTTTCCCACTCTCTTTAAATGTTTT
PF10_0302	28 kDa ookinete surface protein (Pfs28)	AGAAAATGAAGTGTGTACATTAGAAGGAA GATGTATCAGCCTGGTCCACAGT
MAL8.P1.16	Rhomboid protease ROM3 (ROM3)	AAACTTGAGCACACCAAATGTTCAT CGGTAGCACAAGTCTCCAATATTG

^a^Gene identification number from PlasmoDB

^b^Primers designed using the Primer Express Software (Applied Biosystems). Pfs16 and Pfs25 primers designed by Schneider et al. [21]

### Data analysis

Threshold cycle (Ct) values from the measurement of the cDNA samples were obtained in duplicate using the 7000 and the 7500 fast system Software (Applied Biosystem) and further analysed using an excel data sheet (Microsoft Corporation). The average value was taken as the result and only duplicate Ct values within ±1 Ct difference were accepted. Ct values were used to quantify the relative amount of target PCR product present in each reaction; values are inversely proportional to the amount of template in the sample.

Gene expression during gametocytogenesis was represented with an adaptation of the method described by Livak and Schmittgen as (Ct_gene_−Ct_18S rRNA_)T_time ×_ −(Ct_gene_−Ct_18S rRNA_)T_0_, considering the expression at time 0 (T_0_) as the basal expression and using the 18S rRNA gene as housekeeping gene [25]. Absolute quantification (direct comparison of Ct values) was considered the most appropriate method for the drug activity assays.

## Results

### Identification and validation of genes expressed in late-stage gametocytes

In order to confirm expression profiles of genes specific to the later stages of gametocyte development during the gametocytogenesis process [24], a selection of 12 genes previously described in the literature as being gametocyte specific were selected (Table 2). Initially, the transcription profile of those genes throughout a gametocytogenesis process using the qPCR technique was investigated. The experiment was done in duplicate for 15 days, with one arm extended up to 30 days to determine the stability of the mRNAs once mature gametocytes were developed. Results corresponding to the 30-day assay are presented, as similar results were obtained in both assays during the first 15 days. Results were normalized with the housekeeping gene (18S rRNA) and then referenced to time 0. Genes with increasing expression were represented by negative Ct values.

**Fig. 2:**
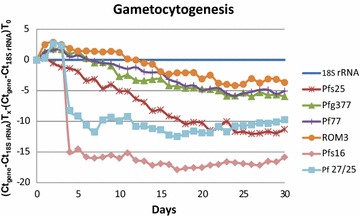
Graphic representation of gene expression throughout the 30 days of gametocytogenesis. *Y axis* shows gene expression represented as (Ct_gene_−Ct_18S__rRNA_)T_time_ × −(Ct_gene_−Ct_18S rRNA_)T_0_, considering the time 0 as the basal expression. Among the 12 genes analysed only the ones shown in the graph had a clear kinetic profile

Although all the 12 selected genes had been described as stage-specific in the literature, only the sub-set of genes coding for Pfs25, ROM3, Pfg377, Pf77, Pfs16, and Pf 27/25 showed a temporal increase in expression during gametocytogenesis when analysed using qPCR (Fig. 2; Additional file 1). Among the genes previously described as specific of early gametocyte stages, only genes coding for proteins Pfs16 and Pf 27/25 showed an increase in expression at day 4 of gametocytogenesis with the majority of the gametocytes at stage I and II (Fig. 2). After day 7, mRNA levels of these genes remained stable. In the case of the genes previously described as specific for the later stages of gametocyte development, only the ones coding for Pfs25, ROM3, Pfg377 and Pf77 were identified as specific for mature gametocytes (Fig. 2). However, the magnitude of the change in expression levels was different among the different genes. ROM3, Pfg377 and Pf77 protein coding genes showed an increase in expression starting around day 10 up to day 20 of approximately 6 Ct (100-fold change when compared to the basal levels at day 0), with expression levels stabilizing after day 20. When using qPCR, a difference of 3 Cts corresponds to an approximately tenfold change in the total amount of cDNA (Additional file 2). In comparison, expression of the Pfs25 protein gene showed a variation of 10 Ct until day 20 before stabilizing (ca. 1000-fold increase).

**Fig. 3:**
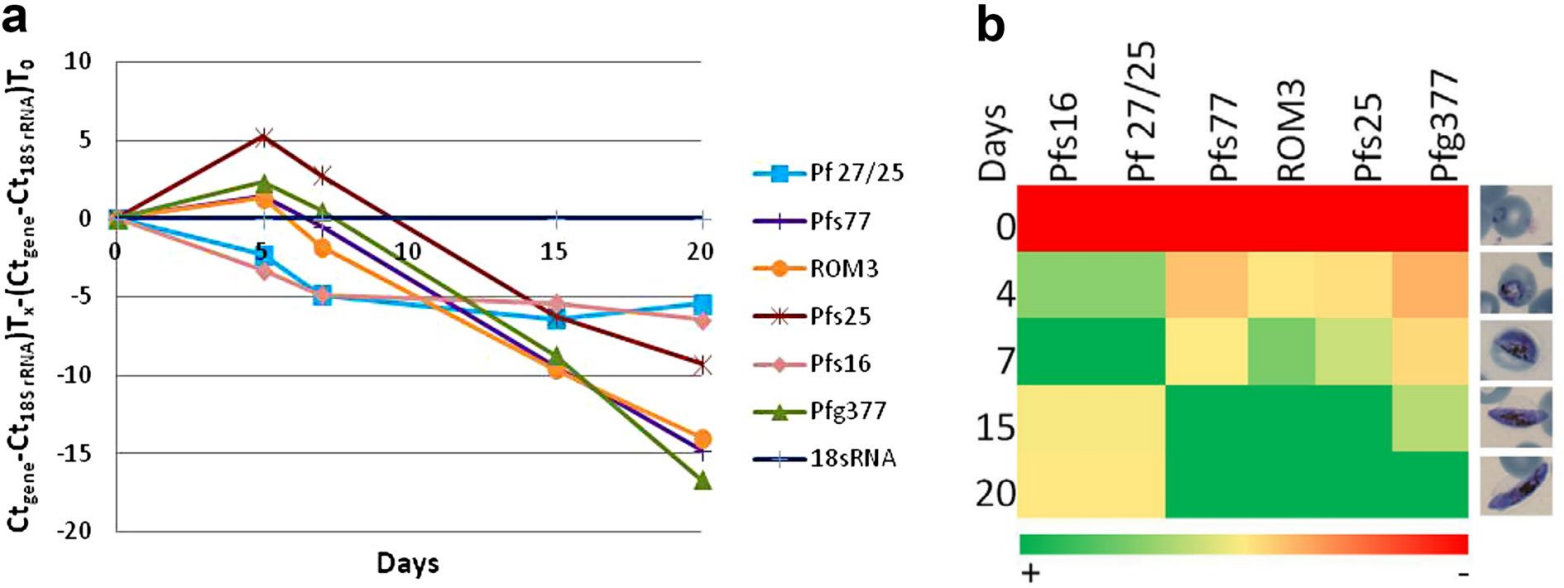
Stage-specific gene expression. Graphic representation of gene expression in gametocytogenesis using bistratene A to remove asexual stages from day 4. Gene expression profile represented: **a** as (Ct_gene_−Ct_18S rRNA_)T_time_ × −(Ct_gene_—Ct_18S rRNA_)T_0_, considering the time 0 as the basal expression; **b** in a heat map with an individual colour scale from the lowest expressed (*red*) to the highest expressed (*dark* *green*) levels

Using standard protocols for the in vitro development of gametocytes, there is a longer co-existence of asexual and sexual stages during the process. Therefore, a new gametocytogenesis protocol was carried out using bistratene A from day 4 until the end of the experiment to remove asexual parasites earlier. The gene expression analysis of these new samples is represented in Fig. 3a, with results confirming what was observed in the previous assay, but with a smaller increase in expression of genes coding for proteins Pfs16 and Pf 27/25 at day 5, because of the earlier elimination of asexual parasites. An additional representation of the data as heat map was also generated (Fig. 3b). Genes expressed in early gametocytogenesis had a maximum expression at day 7 and genes specific to the later stages of gametocyte development (stages IV and V) peaked at days 15–20. This result confirmed that the genes for proteins ROM3, Pfg377, Pf77, and Pfs25 were highly expressed in the later stages of gametocyte development, and could be used for analysing drug gametocytocidal activity.

### Determination of gametocytocidal activity of standard anti-malarial drugs

Gametocytocidal activity of anti-malarial drugs was investigated using a similar method to that previously described for asexual stages [13]. Two different treatments were performed in parallel, one with constant drug pressure throughout the experiment (T_∞_), 144 h in total, and another in which the gametocyte cultures were exposed to the compound for only 48 h, the drug was washed out and then cultured for a further 96 h in fresh complete culture medium (T_48_) (see Fig. 1).

**Fig. 4:**
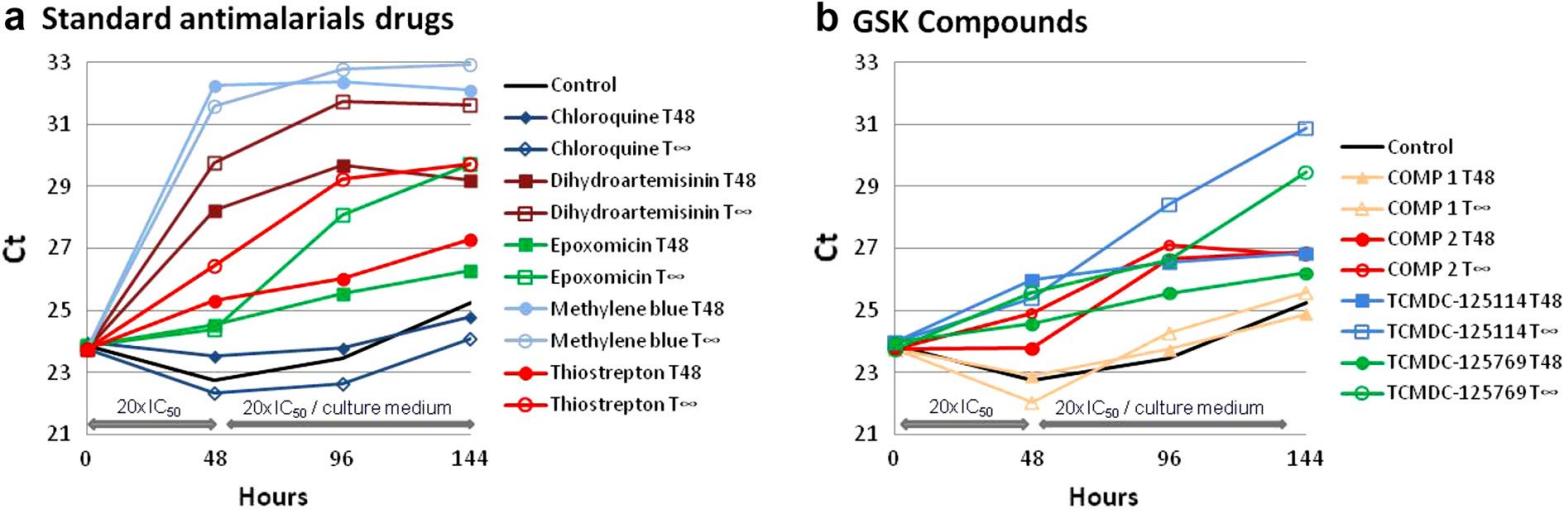
Graphic representation of the Ct values of the Pf77 coding gene after treatment with **a** standard anti-malarial drugs or **b** compounds from the GSK collection. Only protein Pf77 gene is represented, but all genes tested (ROM3 and Pfg377) behaved similarly. Two different protocols were used: T48 corresponds to the cultures where compounds were removed after 48 h and replaced by complete media; T∞ indicates that cultures were maintained with drug pressure throughout the 144 h of the experiment

The assay was validated against standard anti-malarial drugs: epoxomicin (EX), dihydroartemisinin (DHA), chloroquine (CQ), thiostrepton (TS) and methylene blue (MB). Results corresponding to the Pf77 coding gene are presented (Fig. 4a), though profiles for ROM3 and Pfg377 behaved similarly (Additional file 3). As expected, CQ treatment did not affect mature gametocytes and the Ct values were similar to the untreated control, even when CQ treatment was maintained during the whole experiment (Fig. 4a). DHA, TS and EX treatments resulted in an increase in at least 5 Ct values in samples with drug present for 144 h (T_∞_), indicative of a minimum decrease of ca. 100-fold in mRNA levels. However, if drug was only present for 48 h (T_48_), mRNA levels were reduced less than tenfold after drug removal, suggesting a static behaviour of the compounds. In the case of MB, the decrease in mRNA levels initiated during the first 48 h of treatment continued independently of whether constant drug pressure was maintained (T_∞_) or if drug was removed after 48 h of treatment (T_48_), suggesting a cidal effect of the drug. Thus, measurement of specific mRNA levels can be used as surrogate of the activity of compounds acting against later stages of gametocyte development. Furthermore, the differential profile of mRNA expression obtained from constant drug pressure *vs* 48-h exposure could be used to characterize the cidality of the anti-malarial effects of compounds against gametocyte stages.

The assay was further tested against a panel of compounds selected from the GSK compound collection (Table 1). The compounds TCMDC-125520, TCMDC-123475, TCMDC-125133, TCMDC-125769, and TCMDC-125114 have demonstrated anti-malarial activity against asexual stages [23] and against late-stage gametocytes [24]. In addition to these molecules, a close derivative of TCMDC-136869, a quinolone-like compound (COMP 2) displaying an IC_50_ of 0.58 µM against mature gametocytes, was also included in the experiment. As negative control, a close derivative of TCMDC-134278, an azol-like compound (COMP 1) was included, which has no activity against mature gametocytes (IC_50_ >2 µM). Gene expression profiles in response to compound treatment are described in Fig. 4b and Additional files 4 and 5. Compounds TCMDC-125520 and TCMDC-125769 showed similar profiles and data only for TCMDC-125769 are shown in Fig. 4b with the protein Pf77 gene. A constant increase in the Ct values (5 Ct approximately, ca. 100-fold change) was observed when the compounds were maintained throughout the experiment. However, Ct values after drug removal (T_48_) only increased a maximum of 2 Ct (less than tenfold change) indicating a static effect. A similar profile, although with a higher Ct increase (7 Ct, more than 100-fold change), was obtained for compounds TCMDC-125114, TCMDC-123475 and TCMDC-125133 (only TCMDC-125114 is shown in Fig. 4b with protein Pf77 gene). COMP 2 displayed a different expression profile, with an increase of 3 Ct (tenfold change) at 96 h that remained stable independently of whether drug pressure was maintained or withdrawn. Finally, mRNA levels of cultures with compound COMP 1 (negative control) showed no difference compared with the control or the CQ-treated culture, as expected.

In summary, TCMDC-125520, TCMDC-123475, TCMDC-125133, TCMDC-125769, and TCMDC-125114, which were identified as having activity against gametocytes using an ATP gametocytocidal assay [8], showed a prolonged reduction in mRNA levels only when the drug was continuously present during the whole experiment.

### Determination of minimal gametocytocidal concentration

Considering that TCMDC-125114 displayed the strongest effects against mature gametocytes, a new experiment was done to estimate the minimal concentration needed to produce the maximal biological effect against mature gametocytes. This information determined the maximum potential of the compound. Concentrations of TCMDC-125114 at 20 × IC_50_, 60 × IC_50_ and 100 × IC_50_, and for CQ of 2 × IC_50_, 20 × IC_50_ and 125 × IC_50_ values in asexual stages (negative control) were evaluated as described above, with 48 h of drug treatment only. However, a culture treated with 20 × IC_50_ of TCMDC-125114 during the whole experiment (T_∞_) was included as a positive control to achieve inhibition.

**Fig. 5:**
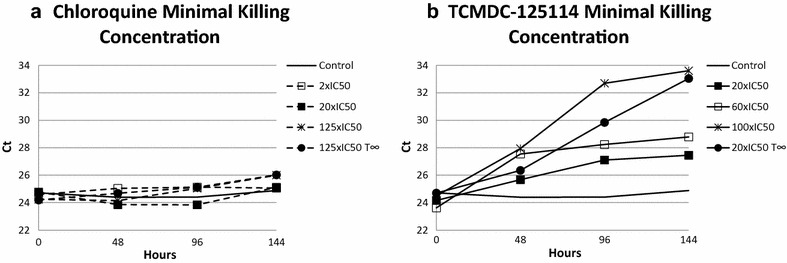
Minimal concentration for cidal effect of TCMDC-125114 with the Pf77 coding gene. With drug exposure for 48 h, increasing concentrations were tested of: **a** chloroquine (negative control); or **b** TCMDC-125114 (active against gametocytes). TCMDC-125114 was used at 20 × IC50 during all the experiments; TCMDC-125114 20 × IC50 with drug exposure throughout the 144 h of the experiment (T∞) was used as a positive control

As expected, no Ct increase was obtained with CQ at any concentration tested (Fig. 5a). In comparison, TCMDC-125114 elicited an increase in the Ct values at 48 h. Increase was proportional to the concentration of drug used (Fig. 5b). At concentrations of 100 × IC_50_, a continuous increase in the Ct values was observed even if drug was removed after the initial 48 h, indicative of cidal activity (Fig. 5b).

## Discussion

Effective treatments aimed at reducing malaria transmission would have a considerable impact on malaria control efforts and significantly reduce the burden of disease. In vitro assays against sexual stages are essential for the identification and characterization of new anti-malarial chemotypes with transmission-blocking activity. In recognition of the extensive work done to produce mature gametocytes in a reproducible manner, the anti-malarial community has now acquired the tools to test anti-malarial drugs against the parasite stage relevant for transmission. Several approaches assessing drug activity against gametocytes have been developed [6, 7, 11, 12, 24, 26]. For example, Blanco et al. [27] recently published an imaging-based HTS assay, based on the ability of mature gametocytes to progress to gametes after drug exposure. The current study described a new assay to support the characterization of any anti-malarial at the drug discovery process that could be used on sexual and asexual stages.

The current study described six genes differentially expressed at specific gametocyte stages, four of which were highly expressed in later stages of gametocyte development. Identification of these genes allowed the discrimination of drug activity against late-stage gametocytes by molecular methods. The use of molecular markers for sex ratio estimation and sexual stage identification has been described previously [18, 22]. Quantification of female and male gametocytes by qPCR relied on the expression of Pfs25 and Pfs230p protein genes for female and male, respectively [22], and the transcriptome of gametocytes from stage I to V was profiled using microarrays [18]. In accordance with previously published studies, the results described in this paper confirm Pfs16 as the most appropriate marker to detect early gametocytogenesis [20, 21] with a profile similar to the one published by Adjalley et al. [11], and expression of ROM3, Pfs25, Pf77, and Pfg377 as the best surrogates to study later stages of gametocyte development [11, 19–21, 27]. Pfs25 was described as expressed exclusively in female mature gametocytes [22], and this is in accordance with the results obtained in this publication, as mixed sexual gametocyte stage cultures were used.

Previously, qPCR has been shown to be a very potent tool, easy to perform and implement, which can be focused on specific parasite stages [19]. In the methods described in this paper, stage-specific mRNA expression levels, determined using qPCR, were used as a surrogate for parasite viability. The method was validated against anti-malarial drugs and research compounds with known activity against gametocytes. Consistent with previous reports, CQ did not show any activity against gametocytes at any of the concentrations tested [28]. For DHA, EX and TS, although there was a reduction in late-stage gametocyte mRNA levels after 48-h of treatment, this effect persisted only when the drugs were present in the culture media. In contrast, with MB the reduction in the gene expression was maintained even after drug removal, indicating gametocytocidal activity, and this is consistent with published data [7, 11]. In fact, MB was reported to be a very effective gametocytocidal agent against gametocyte stages I-V, and has a striking impact on transmission in mosquitoes fed on treated gametocytes [7, 11]. On the other hand, DHA has been reported to be poorly potent against mature sexual stages using an ATP measurement read-out [24], and high concentrations were needed to see an effect. It has been also reported that mature male gametocytes are more sensitive to DHA [26]. The response observed here with DHA could be explained by the concentration used in the assay that corresponded to 20 times the IC_50_ and the methodology used that quantifies only gametocyte mRNA levels. The results obtained with GSK compounds confirmed their activity previously described against gametocytes using an alternative methodology [8, 24]. The majority: TCMDC-125520, TCMDC-123475, TCMDC-125133, TCMDC-125769, and TCMDC-125114, behaved as the commercial anti-malarial drugs TS and EX with the exception of COMP 2 that showed an irreversible effect, although only for an additional 48 h after drug removal. The in vitro gametocytocidal activity of compound TCMDC-125133 and TCMDC-125114 were previously validated ex vivo using the SMFA with a 100 % reduction in the number of oocysts when assayed at 2.5 µM [8].

Dose–response relationships can also be investigated using the described assay, as shown for CQ and TCMDC-125114, and are needed to determine the minimal amount of compound required to produce maximal biological effect. Such studies indicate the biological relevance of findings of anti-gametocyte drug activity, as the potential toxic effects provoked by high compound concentrations must be considered.

## Conclusion

The development of compounds with cidal activity against mature gametocytes is essential for malaria eradication. However, screening for *Plasmodium* transmission-blocking drugs has been very time consuming and typically relied on laborious techniques or expensive devices [11, 24, 27, 29]. This study describes a novel assay for characterizing the activity of anti-malarial drugs against non-engineered unmodified *P. falciparum* late-stage gametocytes using qPCR. The assay analyses the effects of exposing the parasites to a full inhibitory dose of an anti-malarial drug on the mRNA levels of genes specifically expressed in late gametocyte stages. The method described is a reliable and user-friendly technique with a medium throughput that could be easily implemented in any laboratory. Moreover, samples can be stored and processed afterwards, even in a different reference laboratory to where the assays were performed.

## Additional files

10.1186/s12936-016-1429-9 Graphical representation of the expression of 12 selected genes throughout the 30 days of gametocytogenesis. Y-axis shows the gene expression represented as (Ct_gene_−Ct_18S rRNA_)T_time ×_ −(Ct_gene_−Ct_18S rRNA_)T_0_, considering the time 0 as the basal expression. Although only the gametocytogenesis during 30 days is presented, similar results were obtained from day 0 to day 15 in both assays.
10.1186/s12936-016-1429-9 Calibration curve for converting Ct values into fold changes in the total amount of cDNA. Note that when using qPCR, a difference of 3 Cts corresponds to an approximately ten-fold change in the total amount of cDNA.
10.1186/s12936-016-1429-9 Graphic representation of the Ct values after treatment with standard anti-malarial drugs. (A) Pfg377 protein coding gene; (B) ROM3. Two different protocols were used: T_48_ corresponds to the cultures where compounds were removed after 48 h and replaced by complete media; T_∞_ indicates that cultures were maintained with drug pressure throughout the 144 h of the experiment.
10.1186/s12936-016-1429-9 Graphic representation of the Ct values after treatment with compounds TCMDC-125769, TCMDC-125114, COMP 1 and COMP 2 from the GSK collection. (A) Pfg377 protein coding gene; (B) ROM3. Two different protocols were used: T_48_ corresponds to the cultures where compounds were removed after 48 h and replaced by complete media; T_∞_ indicates that cultures were maintained with drug pressure throughout the 144 h of the experiment.
10.1186/s12936-016-1429-9 Graphic representation of the Ct values after treatment with compounds TCMDC-125520, TCMDC-123475 and TCMDC-125133 from the GSK collection with the protein coding genes: (A) Pfs77, (B) Pfg377 and (C) ROM3. Two different protocols were used: T_48_ corresponds to the cultures where compounds were removed after 48 h and replaced by complete media; T_∞_ indicates that cultures were maintained with drug pressure throughout the 144 h of the experiment.
