# Supplementary material for: A novel validated assay to support the discovery of new anti-malarial gametocytocidal agents
Source: Malar J. 2016 Jul 22;15:385. doi: 10.1186/s12936-016-1429-9 (PMC4957904; doi:10.1186/s12936-016-1429-9)
Supplement: Supplementary file 1 — 10.1186/s12936-016-1429-9 Graphical representation of the expression of 12 selected genes throughout the 30 days of gametocytogenesis. Y-axis shows the gene expression represented as (Ctgene−Ct18S rRNA)Ttime × −(Ctgene−Ct18S rRNA)T0, considering the time 0 as the basal expression. Although only the gametocytogenesis during 30 days is presented, similar results were obtained from day 0 to day 15 in both assays. [file 12936_2016_1429_MOESM1_ESM.pptx]

## Slide 1
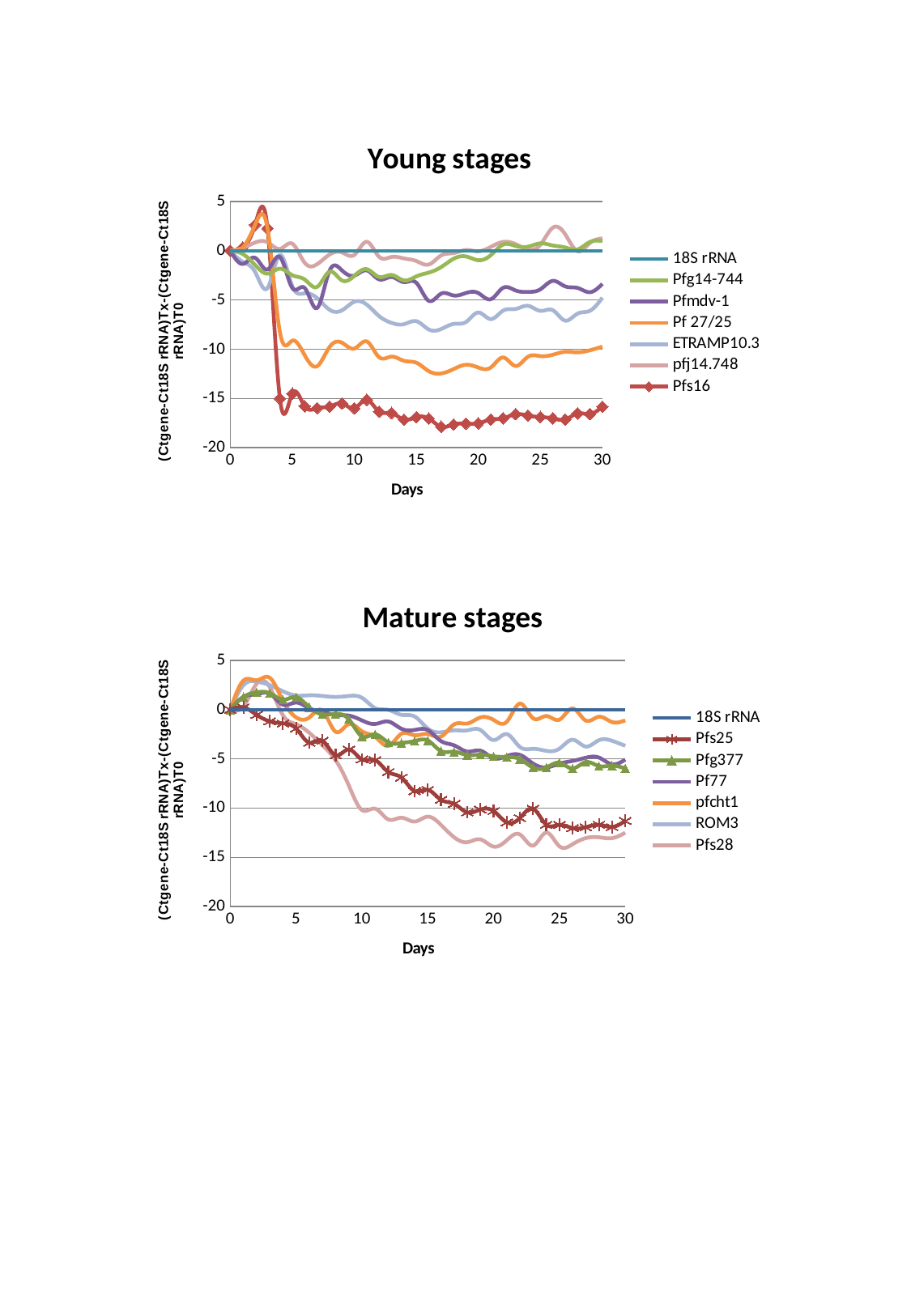

### Chart: Young stages
| Category | Pfs16 | 18S rRNA | Pfg14-744 | Pfmdv-1 | Pf 27/25 | ETRAMP10.3 | pfj14.748 |
|---|---|---|---|---|---|---|---|
### Chart: Mature stages
| Category | 18S rRNA | Pfs25 | Pfg377 | Pf77 | pfcht1 | ROM3 | Pfs28 |
|---|---|---|---|---|---|---|---|
