# Supplementary figures and images for: A novel validated assay to support the discovery of new anti-malarial gametocytocidal agents
Source: Malar J. 2016 Jul 22;15:385. doi: 10.1186/s12936-016-1429-9 (PMC4957904; doi:10.1186/s12936-016-1429-9)

## Slide 1
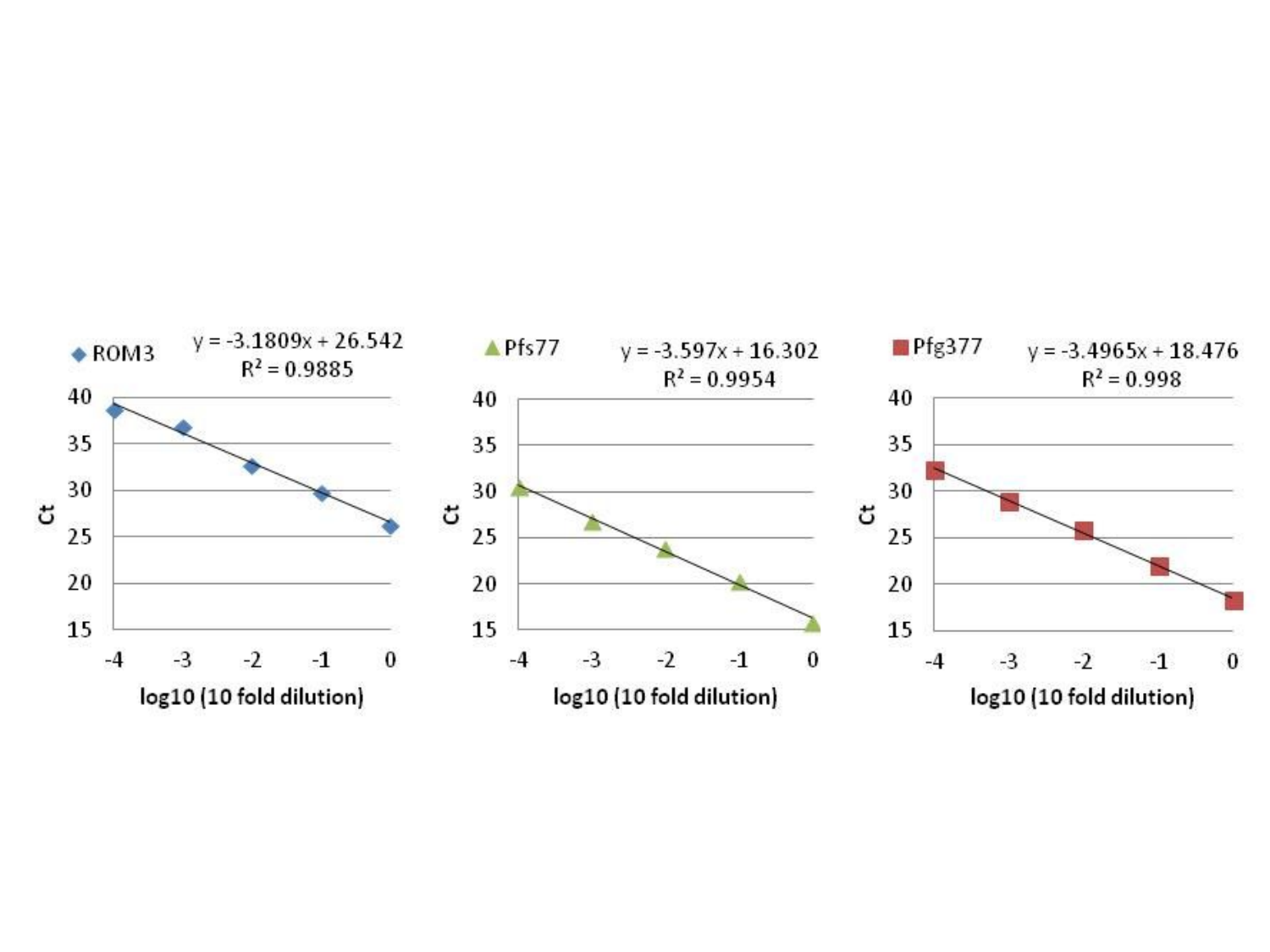

Supplement: Supplementary file 2 — 10.1186/s12936-016-1429-9 Calibration curve for converting Ct values into fold changes in the total amount of cDNA. Note that when using qPCR, a difference of 3 Cts corresponds to an approximately ten-fold change in the total amount of cDNA. [file 12936_2016_1429_MOESM2_ESM.pptx]

## Slide 1
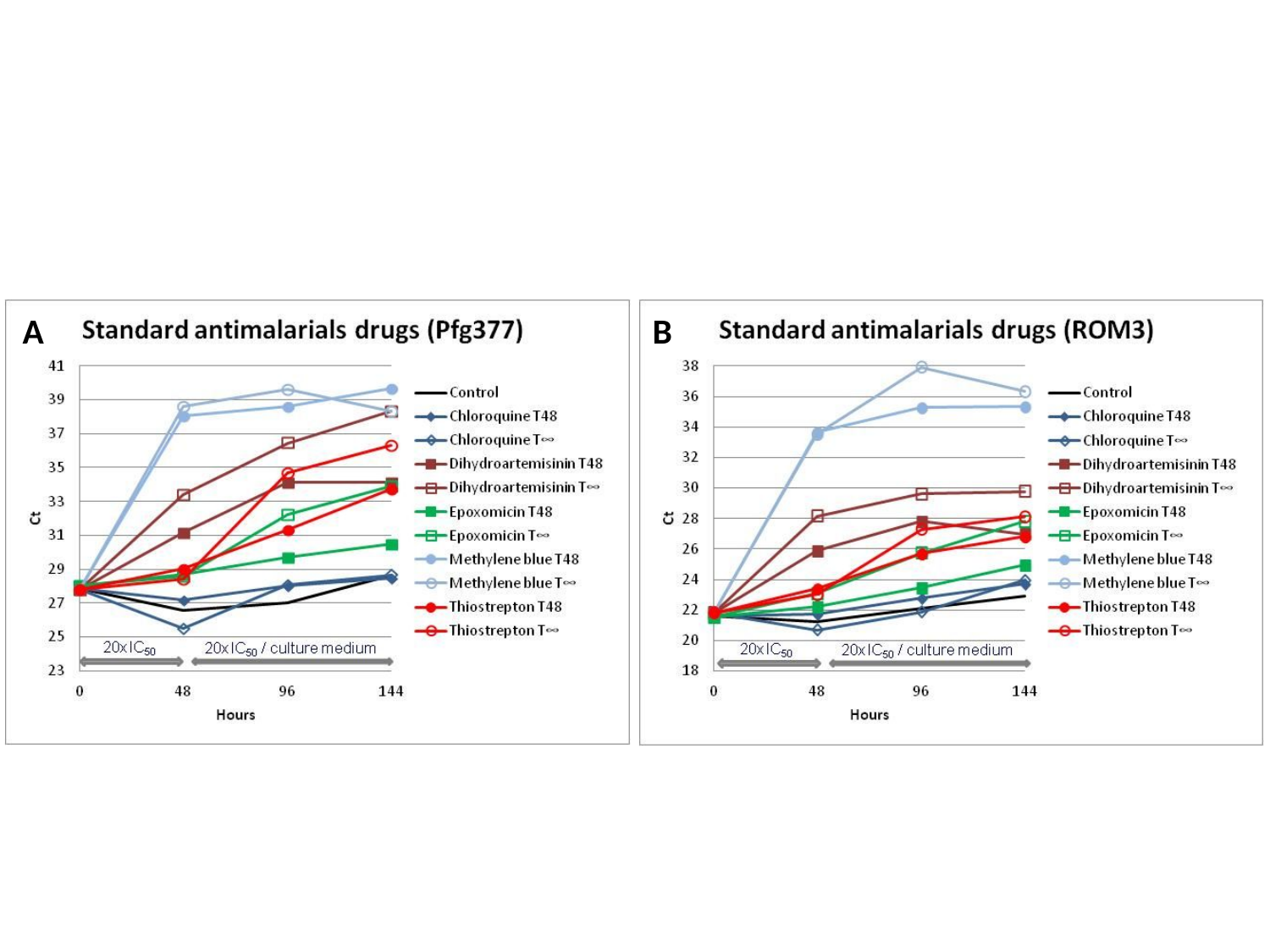

A
B

Supplement: Supplementary file 3 — 10.1186/s12936-016-1429-9 Graphic representation of the Ct values after treatment with standard anti-malarial drugs. (A) Pfg377 protein coding gene; (B) ROM3. Two different protocols were used: T48 corresponds to the cultures where compounds were removed after 48 h and replaced by complete media; T∞ indicates that cultures were maintained with drug pressure throughout the 144 h of the experiment. [file 12936_2016_1429_MOESM3_ESM.pptx]

## Slide 1
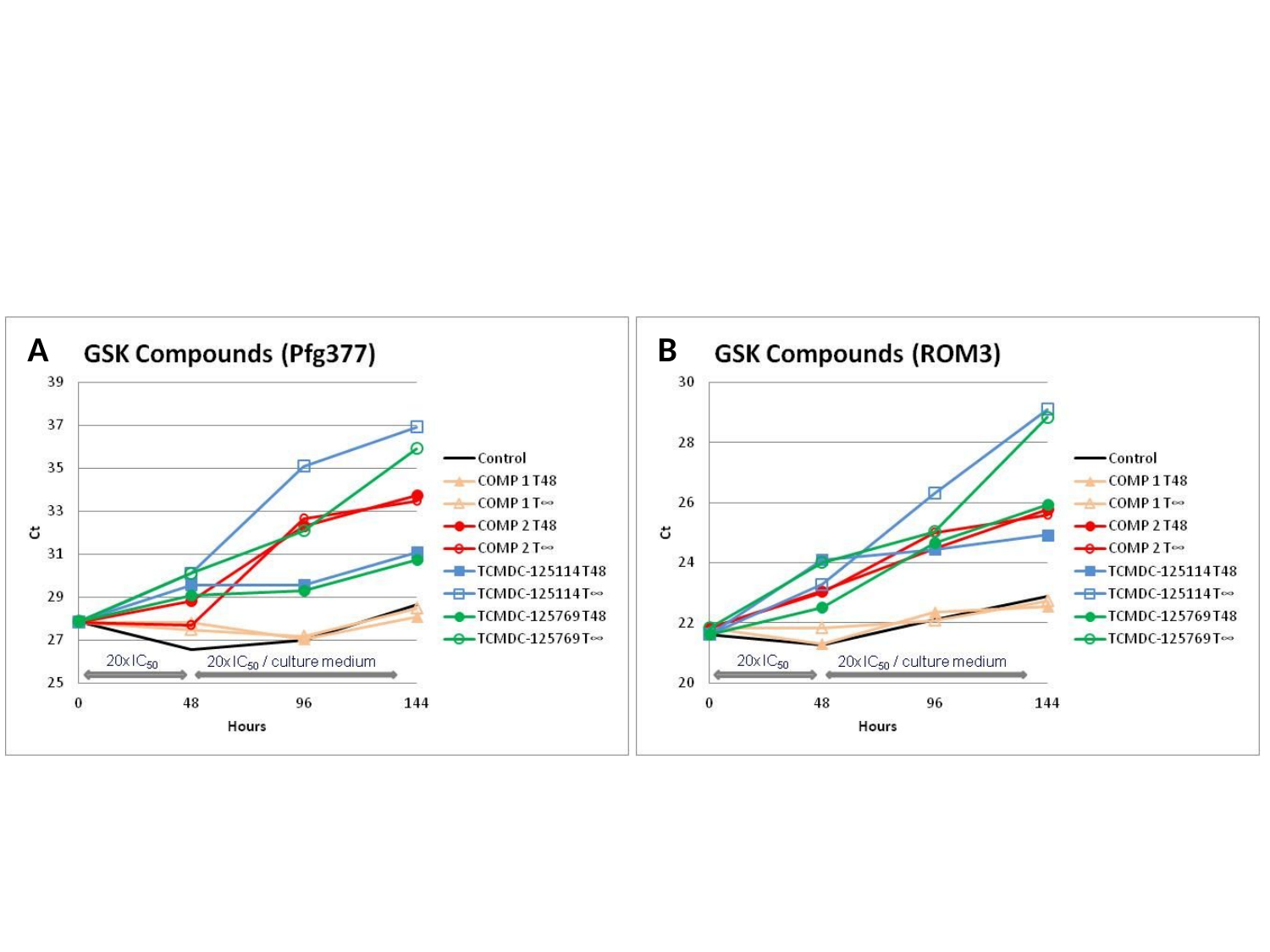

A
B

Supplement: Supplementary file 4 — 10.1186/s12936-016-1429-9 Graphic representation of the Ct values after treatment with compounds TCMDC-125769, TCMDC-125114, COMP 1 and COMP 2 from the GSK collection. (A) Pfg377 protein coding gene; (B) ROM3. Two different protocols were used: T48 corresponds to the cultures where compounds were removed after 48 h and replaced by complete media; T∞ indicates that cultures were maintained with drug pressure throughout the 144 h of the experiment. [file 12936_2016_1429_MOESM4_ESM.pptx]

## Slide 1
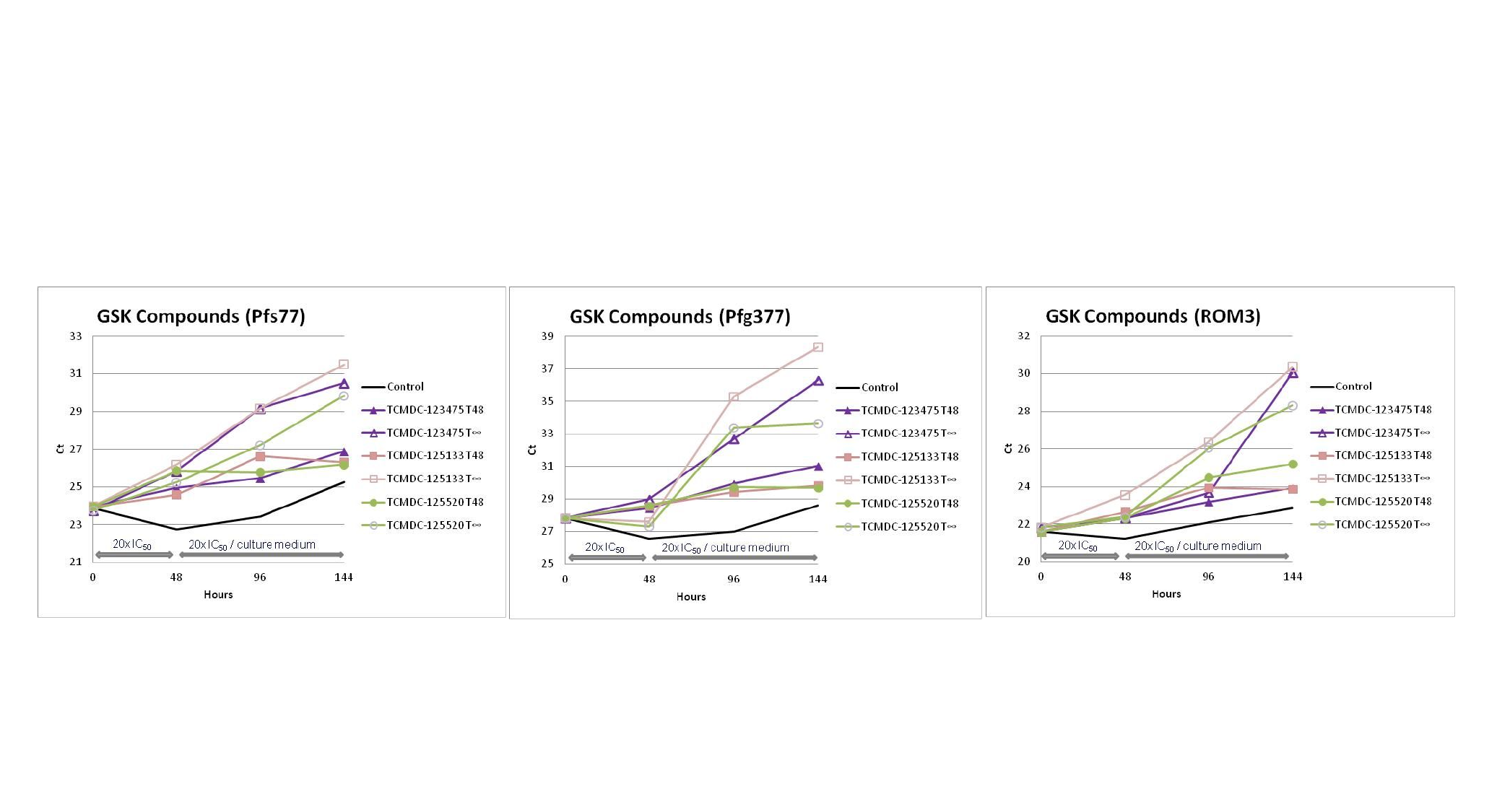

Supplement: Supplementary file 5 — 10.1186/s12936-016-1429-9 Graphic representation of the Ct values after treatment with compounds TCMDC-125520, TCMDC-123475 and TCMDC-125133 from the GSK collection with the protein coding genes: (A) Pfs77, (B) Pfg377 and (C) ROM3. Two different protocols were used: T48 corresponds to the cultures where compounds were removed after 48 h and replaced by complete media; T∞ indicates that cultures were maintained with drug pressure throughout the 144 h of the experiment. [file 12936_2016_1429_MOESM5_ESM.pptx]
